# In Silico–Designed TGFβRI/TGFβRII Receptor Complex Peptide Inhibitors Exhibit Biological Activity In Vitro

**DOI:** 10.1111/jcmm.70548

**Published:** 2025-04-17

**Authors:** Jacek Plichta, Michał Karbownik, Piotr Kuna, Michał Panek

**Affiliations:** ^1^ Department of Internal Medicine, Asthma and Allergy Medical University of Lodz Lodz Poland; ^2^ Department of Pharmacology and Toxicology Medical University of Lodz Lodz Poland

**Keywords:** fibrosis, immunooncology, immunotherapy, in silico, peptide inhibitor, TGF‐β, tumour microenvironment

## Abstract

TGF‐β (transforming growth factor β) is a pleiotropic cytokine found in three isoforms in humans. It regulates cell proliferation, wound healing, immune cell recruitment, contributes to epithelial‐to‐mesenchymal transition (EMT) and to the conversion of fibroblasts to myofibroblasts. TGF‐β signalling pathway hyperactivity underlies many human disorders. The aim of this study was to evaluate a series of novel, in silico–designed peptide inhibitors (PIs) of the TGFβ/TGFβRI/TGFβRII complex. Luciferase‐based luminescence assays on HEK293T cells were used to comparatively assess PI biological activity and calculate IC_50_ values. Flow cytometry was used to assess PI cytotoxicity on HEK293T cells. The PIs caused significant luminescence level reductions compared to controls. Additionally, three of the PIs caused luminescence reductions that did not differ significantly from the effects of SD‐208, a small molecule TGFβ inhibitor. None of the PIs exhibited cytotoxicity. Our TGFBR PIs have demonstrated activity in vitro, with no observed cytotoxicity. Our results suggest the PIs may be of interest in the treatment of fibrotic disorders, chronic inflammatory diseases, or certain neoplastic cancers. The PIs will be further refined in silico and tested via assays carried out on cancer cell lines and CD4+/CD8+ T cells.

## 1 Introduction

TGF‐β (transforming growth factor β) is a pleiotropic cytokine found in three isoforms (TGFβ1, TGFβ2, TGFβ3) in humans. It regulates cell proliferation, contributes to epithelial‐to‐mesenchymal transition (EMT), contributes to the conversion of fibroblasts to myofibroblasts and causes overproduction of the extracellular matrix (ECM) in tissues undergoing fibrosis. TGF‐β upregulates the expression and synthesis of many matrix proteins, primarily through the recruitment of myofibroblasts [1]. Additionally, in the early stages of fibrosis, TGF‐β stimulates myofibroblasts and other stromal cells to enhance the synthesis of collagen crosslinking enzymes, particularly lysyl oxidase, which increases the rigidity of the collagen network. Moreover, TGF‐β downregulates the synthesis of matrix‐depleting proteins, such as matrix metalloproteinases (MMP‐1, ‐8, ‐13), leading to a decrease in the rate of ECM degeneration [2]. Cumulatively, these effects may lead to a fibrotic response in affected areas, such as asthmatic airways or tumour microenvironments [3]. The TGF superfamily of proteins (and especially TGFβ1) is also responsible for immunosuppression of T and B lymphocytes, as well as NK cells, and chemotaxis of macrophages and fibroblasts [4]. Additionally, TGF‐β inhibits MHC class II antigen expression and type II pneumocyte surfactant synthesis [5]. TGF‐β receptors (TGFβRs) are expressed by all human cell types [6]. Seven subtypes of type I TGF‐β receptors can be distinguished (activin receptor‐like kinases 1–7, ALK1‐ALK7). Five type II receptors (ActRIIA, ActRIIB, BMPRII, AMHRII and TβRII) are present [7]. TGF‐β cytokines, first of all, bind to TGFβRII (ALK1), which leads to the recruitment of TGFβRI (ALK5) and the subsequent formation of a TGFβRI/TGFβRII dimer [8]. This complex is responsible for phosphorylating TGFβRI kinase domains, resulting in activation of the SMAD pathway [9]. Activation of TGFβRI/TGFβRII through the binding of TGF‐β ligands stimulates the canonical signalling pathway via the formation of Smad complexes, which translocate to the nucleus, where they act as transcription factors. This process may also take place via non‐Smad pathways, in which the Erk1/2, JNK and p38 MAP kinase pathways, the Src tyrosine kinase, phosphatidylinositol 3'‐kinase and Rho GTPases are also involved [10]. The SMAD protein family and MAP kinases are the primary intracellular effectors of TGF‐β signalling [11]. ALK5 (TGFβRI), if activated by directly binding TGF‐β ligands, induces MAPK, TAK1, JNK, ERK1/2 and p38 activation in the cell, which influences target gene expression independently of SMAD proteins [12]. The conditions for differential activation of ALK1 and ALK5 signalling pathways vary between tissues and cell types and depend strongly on the presence of specific and non‐specific activation‐inducing factors [13]. Evidence increasingly points towards the varied responses to TGF‐β signalling stemming from regulatory crosstalk between the several pathways, which include TGF‐β as a component. The pathways involved often share receptors, transcription factors and ligands, while retaining discrete effects on the transcription of target genes [14, 15].

**FIGURE 1:**
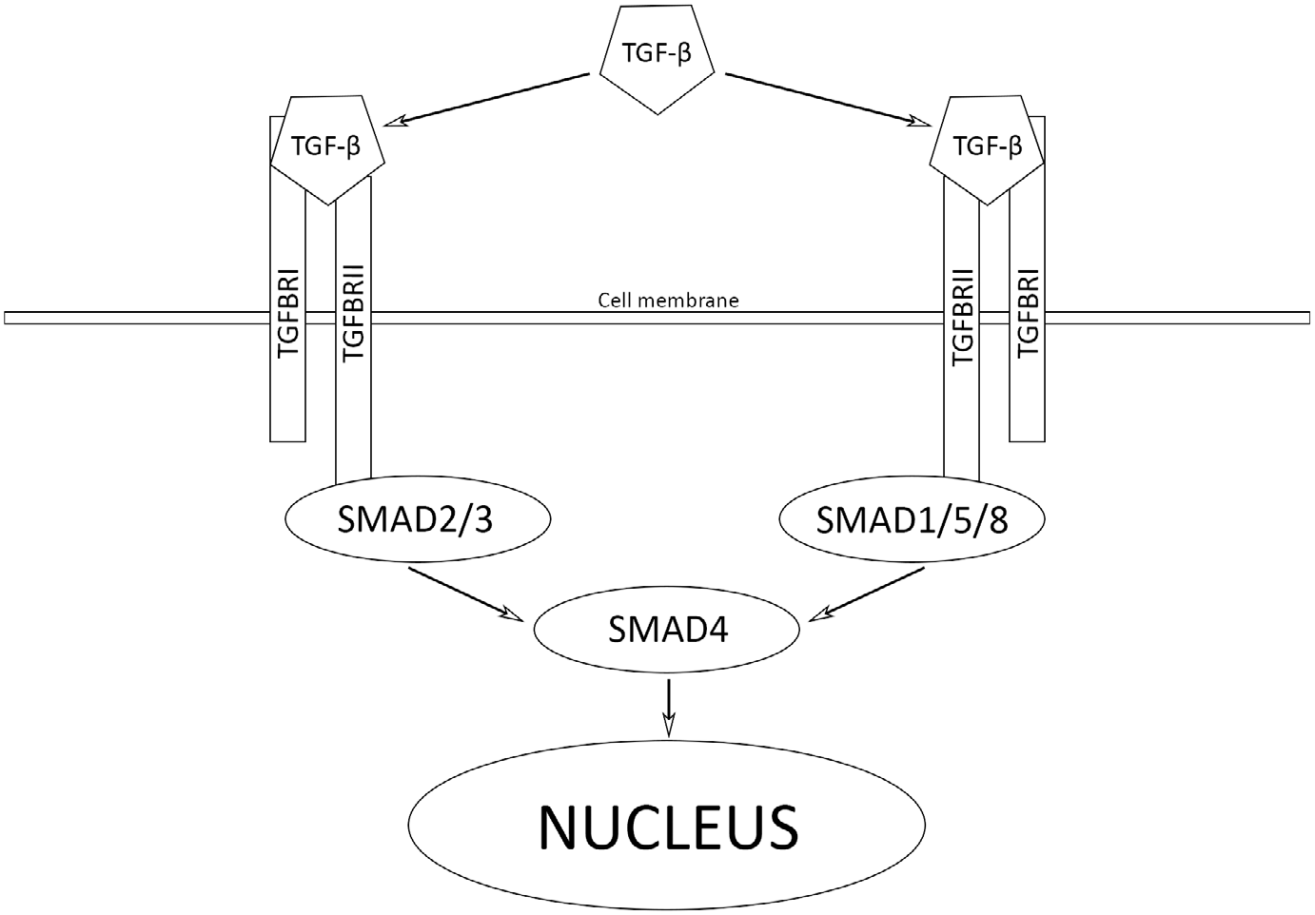
Signalling scheme of the TGFβ‐SMAD signalling pathway. TGF‐β is initially secreted in an inactive form and activated in an integrin‐dependent manner. Activated TGF‐β ligands bind TGF‐β type II receptors (TGFβRII), transmembrane receptors with serine/threonine kinase activity. Interactions between TGF‐β and TβRII cause recruitment and phosphorylation of TGF‐β type I receptors (TGFβRI). Activated TGFβRIs phosphorylate SMAD2 and SMAD3, which form heterodimers, and then trimers with SMAD4. SMAD protein complexes translocate to the nucleus and influence the TGF‐β target gene expression.

Figure 1 depicts the general signalling scheme of the TGFβ‐SMAD signalling pathway.

Hyperactivity within the TGFβ‐SMAD signalling pathway underlies many human disorders, such as fibrotic disorders, progressive cancers and excess deposition of ECM. Patients with asthma, chronic obstructive pulmonary disease, idiopathic pulmonary fibrosis, renal failure, heart failure, myocardial infarction, cystic fibrosisand systemic scleroderma exhibit elevated TGF‐β expression [16, 17, 18]. Furthermore, the deregulation of TGF‐β cell signalling is the main mechanism responsible for initiating and regulating the progression of fibrosis in various tissues, including the neoplastic tissues of immune‐excluded tumours. In several cancers, including some lung and kidney cancers, TGFβ‐derived fibrosis creates a physical barrier between the tumour and immune cells [19]. TGF‐β signalling dysregulation can also drive cancer metastasis. In homeostatic tissues and early carcinogenesis, TGF‐β activity drives inflammatory responses and the apoptosis of damaged cells. However, when tumours progress, TGF‐β activity results in an unrestrained wound‐healing reaction in cancer‐associated fibroblasts and suppresses the innate and adaptive immune responses [20]. This ultimately means that in cancer development, TGF‐β has a dual, ‘paradoxical’ role. In healthy epithelial cells and early carcinogenesis, TGF‐β suppresses tumour growth by inducing the expression of CDK inhibitors p15INK4B and p21CIP1, arresting the cell cycle and promoting apoptosis. In later stages of malignancy, many tumours become resistant to the suppressive effects of TGF‐β due to loss of function mutations in components of the PI3K/AKT and RAS/MAPK pathways [21]. However, tumour cells commonly maintain functional TGF‐β/SMAD pathways, the hyperactivity of which in turn promotes epithelial–mesenchymal transition (EMT) and metastasis. TGF‐β also activates the transcription factors SNAIL1/2, ZEB1/2 and TWIST1/2, which promote EMT in a SMAD‐dependent manner [22]. Furthermore, TGF‐β suppresses the activity of dendritic cells, macrophages, natural killer cells, CD4+ and CD8+ cells. TGF‐β also stimulates the proliferation of Treg cells, which further downregulate the immune response. What is more, TGF‐β downregulates genes responsible for cytotoxic T lymphocyte (CTL)–mediated tumour cytotoxicity, further assisting tumour progression [23]. TGF‐β seems to be a key player in the reprogramming of the tumour microenvironment (TME), and peer‐reviewed data demonstrate that TGF‐β inhibitors may restore immune responses to cancer [24]. This novel approach can synergise with other immunotherapies like immune checkpoint inhibitors, CAR‐T, or cancer vaccines to enhance the anti‐tumour immune response.

The aim of this study was to conduct a preliminary evaluation of the efficacy of novel peptide inhibitors, which upon binding to the TGFβRI/TGFβRII receptor, would prevent the TGF‐β ligand from binding and competitively inhibit the formation of the activating complex, ultimately blocking the activation of TGF‐β signalling pathways.

## 2 Methods

### 2.1 Identification of the Minimal TGF‐β–TGFβRI and TGF‐β–TGFβRII Interface, Peptide Library Creation, Peptide Inhibitor Synthesis

Interface regions between the TGF‐β ligand and its receptors (TGFβRI, TGFβRII) were identified based on manual examination of the TGFβRI/TGFβRII/TGF‐β complex (Protein Data Bank, PDB: 3KFD: Ternary complex of TGF‐β1 reveals isoform‐specific ligand recognition and receptor recruitment in the superfamily, https://www.wwpdb.org). The newly identified interface regions were segmented into peptides made of 9–12 amino acids, and a single amino acid shift was introduced to each segment. This method of segmentation was utilised in the creation of a primary peptide inhibitor (PI) library.

This library was then utilised in in silico docking assays for the TGFβRI/TGFβRII/TGF‐β complex using the HADDOCK (High Ambiguity Driven protein–protein DOCKing, https://wenmr.science.uu.nl/haddock2.4) and CABS‐Dock software (CABS‐dock server for flexible protein‐peptide docking, https://www.wwpdb.org). Docking test results were manually validated and used as the basis for the selection of peptides viable for further biological activity research.

**FIGURE 2:**
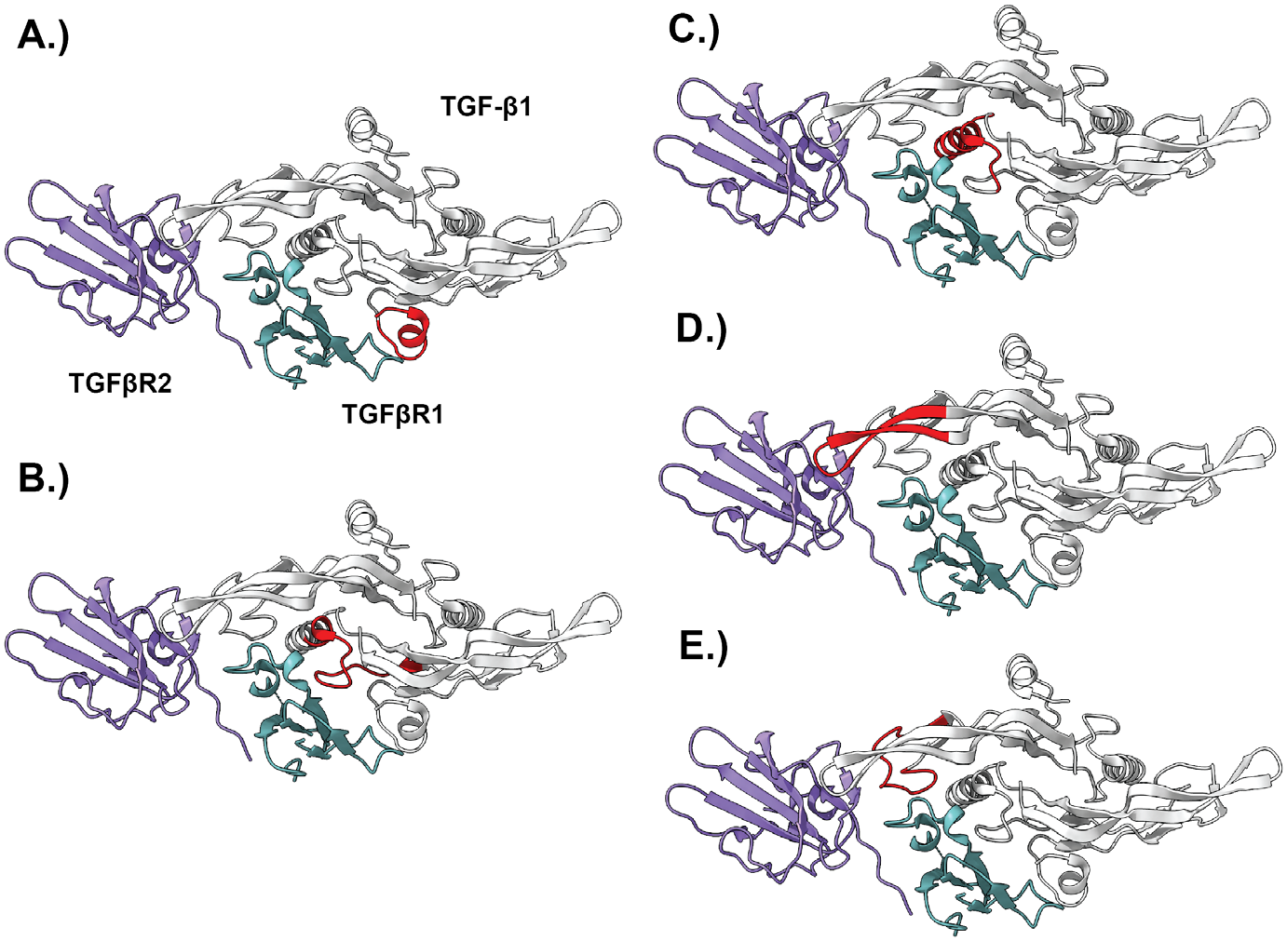
Interface regions used to design the novel peptide inhibitors, marked in red on the crystallography structure of the TGFβ‐TGFβRI/TGFβRII complex. Amino acid sequences for the interface regions used to design the novel inhibitors were as follows: (A) ALDAAYCFRNVQD; (B) CAGACPYLWSSDTQHSR; (C) SDTQHSRVLSLYNTINPEAS; (D) LPIVYYVGRKPKVEQL; (E) RKDLGWKWIHEPKGY.

The newly designed peptide inhibitors were synthesised by GenScript Biotech Corporation, 860 Centennial Ave, Piscataway, NJ 08854, USA. All examined interface regions were visualised in Figure 2.

### 2.2 Non‐Peptide Small Particle TGF‐β Inhibitors SD‐208 and SB‐525334

For comparative analyses of inhibitor biological activity, commercially available inhibitors SD‐208 (2‐(5‐Chloro‐2‐fluorophenyl)pteridin‐4‐yl]pyridin‐4‐yl‐amine), (cat. no. 627536‐09‐8) and SB‐525334 (6‐[2‐tert‐Butyl‐5‐(6‐methyl‐pyridin‐2‐yl)‐1H‐imidazol‐4‐yl]‐quinoxaline) (cat. no. 356559–20‐1), both produced by MERCK, were used.

SD‐208 is a small‐molecule, selective inhibitor of TGFβRI. It prevents SMAD2 phosphorylation by binding to the receptor kinase and maintaining it in an inactive configuration [25]. This inhibitor was employed as a reference in this study due to its well‐established pharmacodynamic properties across multiple studies. It has been demonstrated to inhibit TGF‐β1 and TGF‐β2 cytokine‐stimulated cell growth and migration, as well as to reduce the effects of these cytokines on the decreased susceptibility of malignant tumour cells to the immune system, both in vivo and in vitro [26]. SD‐208 also inhibits the effects of TGF‐β1 on physiologically relevant cells, such as vascular endothelial smooth muscle cells, reducing their proliferation and migration, and smooth muscle and goblet cells in the airways, diminishing their TGF‐β1‐induced proliferation in response to allergens [27].

SB‐525334 is the second reference inhibitor for TGFβRI used in this study. It has demonstrated selective inhibition of TGF‐βRI and an inhibitory effect on the phosphorylation and nuclear translocation of SMAD signalling proteins, as well as on TGF‐β1‐induced gene transcription [28]. These pharmacodynamic properties allowed SB‐525334 to exhibit inhibitory effects on carcinogenesis in an ovarian cancer cell model, reducing the proliferative potential of cancer cells and their ability to migrate and invade [29].

### 2.3 Comparative Assessment of TGF‐β Inhibitor Biological Activity Using Luciferase‐Based Luminescence Assays on HEK293T Cells

The human embryonic kidney cell line HEK293T CAGA12‐Luc/TK Renilla (Ximbio, cat. no 156438) used in this study was modified for use as a reporter cell line for measuring TGF‐ β signalling. The cell line stably expresses CAGA12‐Luc, a Smad3/Smad4‐dependent reporter containing 12 copies of CAGAC motifs derived from the PAI‐1 promoter. This reporter protein is expressed and secreted by HEK293T cells upon TGFβRI/TGFβRII activation. The cell line also contains a Renilla reporter driven by a thymidine kinase (TK) promoter that acts as an internal control.

The presence of the described reporter activity allows HEK293T cells to be used to measure TGF‐β signalling activity using luciferase assays. The Promega Dual‐Luciferase Reporter Assay System (Promega, cat. no E1910) kit was used for assays on the HEK293T cell line. The following procedure was carried out according to manufacturer protocols. Cells were seeded in 96‐well plates coated with poly‐d‐lysine (PDL), in a DMEM 10% FBS medium. All experiments were carried out with the use of 4 biological replicates per group. After 24 h of incubation, the culture medium was removed and the cells were washed three times with PBS. Then, fresh culture medium with either a commercial small molecule inhibitor (SD‐208 or SB‐525334), always at a final concentration of 20 μM, or a novel peptide TGF‐β inhibitor (concentrations from 500 nM to 50 μM) was added, and the cells were incubated for 60 min. After incubation, an additional volume of fresh blank medium or medium supplemented with TGF‐β was added to the wells. The cells were incubated again for 6 h, after which the contents of the wells were removed and the wells rinsed again with PBS. The cells were stored at −80°C until measurement.

Prior to measurement, cells were lysed using a passive lysis buffer. The lysate from each well was added to corresponding wells on a new 96‐well plate containing a reagent that induces luminescence of the first reporter protein, which is expressed by the HEK293T cells upon TGF‐β receptor activation. Luminescence was read using a plate reader with a luminescence measurement function (Victor EX, Perkin Elmer), following the kit manufacturer's instructions. After the measurement, a reagent that activates the second reporter protein (constitutively expressed by HEK293T cells, serves as an internal control) was added to each well, and the luminescence reading was repeated. All luciferase‐based assays were conducted using 4 biological replicates (cell cultures seeded and cultured independently) for each inhibitor and concentration tested.

### 2.4 Assessment of PI Cytotoxicity via Flow Cytometry on HEK293T Cells

HEK293T cells were seeded in 12‐well plates coated with poly‐d‐lysine (PDL) in a DMEM 10% FBS medium. After 24 h of incubation, the culture medium was removed and the cells were washed three times with PBS. Next, either empty fresh culture medium, medium with the addition of a commercial small molecule (SD‐208 or SB‐525334), or one of the novel peptides TGF‐β inhibitors was added. After 24 h of incubation, cells were removed from the wells and counted using a haemocytometer.

Cells were prepared for flow cytometry using the Pharmingen FITC Annexin V Apoptosis Detection Kit I (BD Biosciences, cat. no 556547). 100,000 cells from each sample were suspended in a binding buffer and then stained with FITC and/or propidium iodide. After a 15‐min incubation, the fluorescence profiles of each cell population were measured using a flow cytometer (CytoFLEX, Beckman Coulter), identifying the proportion of cells in apoptosis and necrosis. The data were visualised and quantified by constructing a dot plot using the CytExpert (Beckman Coulter) software.

### 2.5 Statistical Analysis

IC50 values were calculated using 4‐parameter logistic regression in the Prism 9.0 (GraphPad) software. The Statistica 13.1 (StatSoft) software was used to carry out a series of two‐sided unpaired Student's t‐tests to compare the impact of the tested inhibitors on the biological activity of TGFβRI/TGFβRII. All measured results were considered significant at α level = 0.05. The α level is the threshold value against which *p*‐values are measured. *p* < 0.05 was considered to indicate a statistically significant difference.

## 3 Results

### 3.1 Peptide Inhibitor Design and Synthesis

In the preliminary studies, five interface regions between TGF‐β and TGFβRI and TGFβRII receptors have been determined [based on the available crystallographic structure of the TGFβRI, TGFβRII, TGFβ (PDB: 3KFD) complex]. On this basis, a primary peptide library has been created in silico (See Table 1). Using the CABS‐Dock and HADDOCK software, all the peptides were designed to dock to an appropriate receptor.

**TABLE 1:** Amino acid sequences for the novel peptide inhibitors of TGFβRI/TGFβRII receptor complex designed for the 5 identified critical binding regions.

Region (Amino acid sequence)	Peptide inhibitors (Amino acid sequence)
1 (ALDAAYCFRNVQD)	1_1 (ALDAAYCFR), 1_2 (LDAAYCFRN), 1_3 (DAAYCFRNV), 1_4 (AAYCFRNVQ), 1_5 (AYCFRNVQD)
2 (CAGACPYLWSSDTQHSR)	2_1 (CAGACPYLW), 2_2 (AGACPYLWS), 2_3 (GACPYLWSS), 2_4 (ACPYLWSSD), 2_5 (CPYLWSSDT), 2_6 (PYLWSSDTQ), 2_7 (YLWSSDTQH), 2_8 (LWSSDTQHS), 2_9 (WSSDTQHSR)
3 (SDTQHSRVLSLYNTINPEAS)	3_1 (SDTQHSRVLSLYNT), 3_2 (DTQHSRVLSLYNTI), 3_3 (TQHSRVLSLYNTIN), 3_4 (QHSRVLSLYNTINP), 3_5 (HSRVLSLYNTINPE), 3_6 (SRVLSLYNTINPEA), 3_7 (RVLSLYNTINPEAS)
4 (LPIVYYVGRKPKVEQL)	4_1 (LPIVYYVGRK), 4_2 (PIVYYVGRKP), 4_3 (IVYYVGRKPK), 4_4 (VYYVGRKPKV), 4_5 (YYVGRKPKVE), 4_6 (YVGRKPKVEQ), 4_7 (VGRKPKVEQL)
5 (RKDLGWKWIHEPKGY)	5_1 (RKDLGWK), 5_2 (KDLGWKW), 5_3 (DLGWKWI), 5_4 (LGWKWIH), 5_5 (GWKWIHE), 5_6 (WKWIHEP), 5_7 (KWIHEPK), 5_8 (WIHEPKG), 5_9 (IHEPKGY)

The preliminary results concerning the design and synthesis of inhibitors for TGFβRI/TGFβRII allowed us to identify five crucial interface regions for TGFβ‐TGFβRI/TGFβRII (Region 1 ALDAAYCFRNVQD, Region 2 CAGACPYLWSSDTQHSR, Region 3 SDTQHSRVLSLYNTINPEAS, Region 4 LPIVYYVGRKPKVEQL and Region 5 RKDLGWKWIHEPKGY). For Region 1, the following peptide inhibitors have been synthesised: 1.1 ALDAAYCFR, 1.2 LDAAYCFRN, 1.3 DAAYCFRNV, 1.4 AAYCFRNVQ and 1.5 AYCFRNVQD. For Region 2, the following peptide inhibitors have been synthesised: 2.1 CAGACPYLW, 2.2 AGACPYLWS, 2.3 GACPYLWSS, 2.4 ACPYLWSSD, 2.5 CPYLWSSDT, 2.6 PYLWSSDTQ, 2.7 YLWSSDTQH, 2.8 LWSSDTQHS, 2.9 WSSDTQHSR. Table 1 shows the amino acid sequences of the peptide inhibitors designed for Regions 1–5.

### 3.2 Assessment of Peptide Inhibitor Biological Activity—Luciferase Assays

The biological efficacies of two commercial small molecule inhibitors (SD‐208 and SB‐525334) of the type I receptor for TGF‐β have been verified, and these inhibitors have been regarded as positive controls for TGFBRI/TGFBRII inhibition in our subsequent measurements. Compared to positive controls (cells incubated with TGF‐β, without the addition of inhibitors), both SD‐208 and SB‐525334 reduced luminescence by approximately 95%.

**FIGURE 3:**
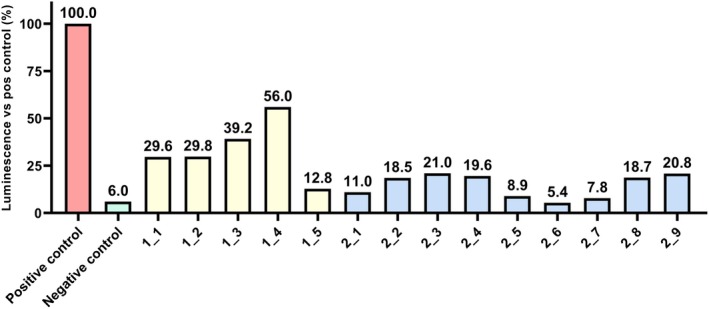
Luminescence values expressed as percentages of the luminescence values exhibited by positive controls (HEK293T cells incubated with TGF‐β, without the addition of inhibitors), for negative controls (naïve HEK293T cells) and HEK293T cells incubated with 50 μM peptide inhibitors 1_1‐1_5 and 2_1‐2_9.

**FIGURE 4:**
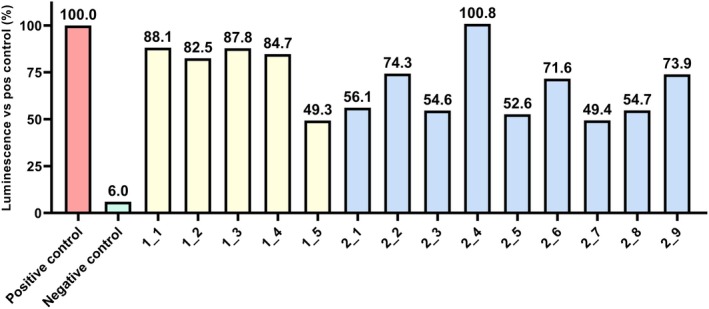
Luminescence values expressed as percentages of the luminescence values exhibited by positive controls (HEK293T cells incubated with TGF‐β, without the addition of inhibitors), for negative controls (naïve HEK293T cells) and HEK293T cells incubated with 20 μM peptide inhibitors 1_1–1_5 and 2_1–2_9.

A series of unpaired t‐tests was carried out using mean luminescence values generated from Dual‐Luciferase Reporter Assays to evaluate and compare the impact of the novel peptide inhibitors on the biological activity of TGFBRI/TGFBRII. In these tests, compared to positive controls (cells incubated with TGF‐β, without the addition of inhibitors), both 20 μM SD‐208 and each of the tested novel peptide inhibitors at 20 and 50 μM were shown to cause a statistically significant (*p* < 0.05) reduction in luminescence. Compared to positive controls, the PIs caused a luminescence level reduction of 80% at 50 μM on average and a reduction of 35% at 20 μM. Additionally, a comparison of mean luminescence values between 20 μM SD‐208 and each of the tested novel peptide inhibitors at 50 μM has shown no statistically significant difference between the effect of 20 μM SD‐208 and peptides 2_5 (*p* = 0.706626), 2_6 (*p* = 0.158059) and 2_7 (*p* = 0.536196) at 50 μM. Figures 3 and 4 show a comparison of luminescence values exhibited by the controls and cells incubated with 50 μM and 20 μM PIs, respectively. The full data set for these statistical tests can be found in Tables S1–S4.

Luminescence data from the luciferase assays was also used to calculate IC50 values for each PI, using 4‐parameter logistic regression. Figures 5 and 6 show charts of luminescence values (relative luminescence units) versus log concentration (μM) for PIs 1_5 and 2_3. Charts for the remaining PIs can be found in Supporting Information (Figures S1–S12).

**FIGURE 5:**
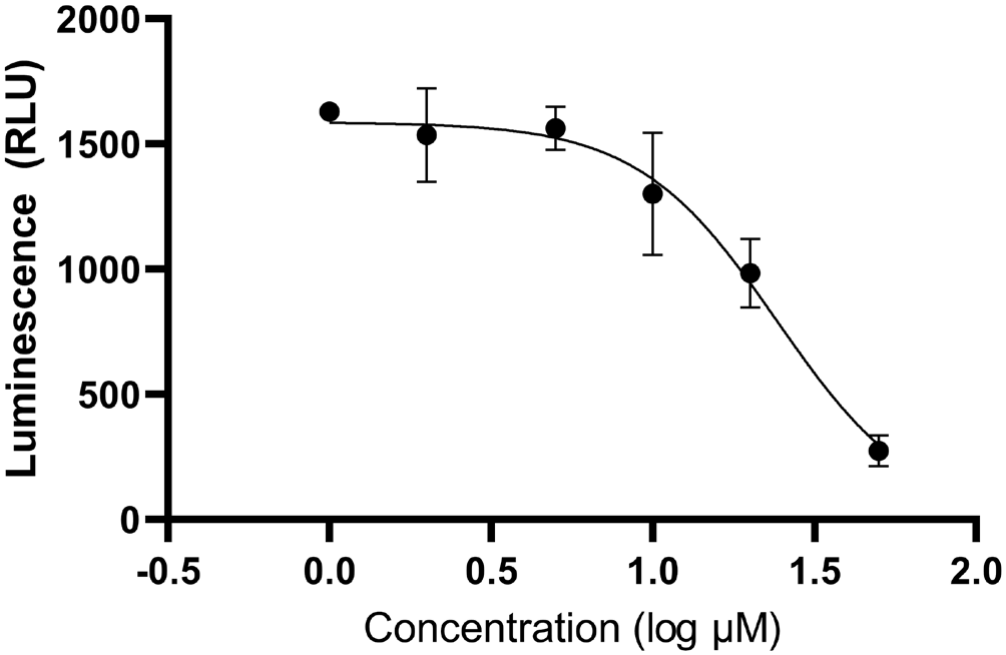
Luminescence (relative luminescence units) against concentration (log μM) of peptide inhibitor 1_5. The IC50 value for this PI was estimated to be 24.21 μM using 4‐parameter logistic regression. Four biological replicates were tested for each concentration. Data represent mean ± SEM.

**FIGURE 6:**
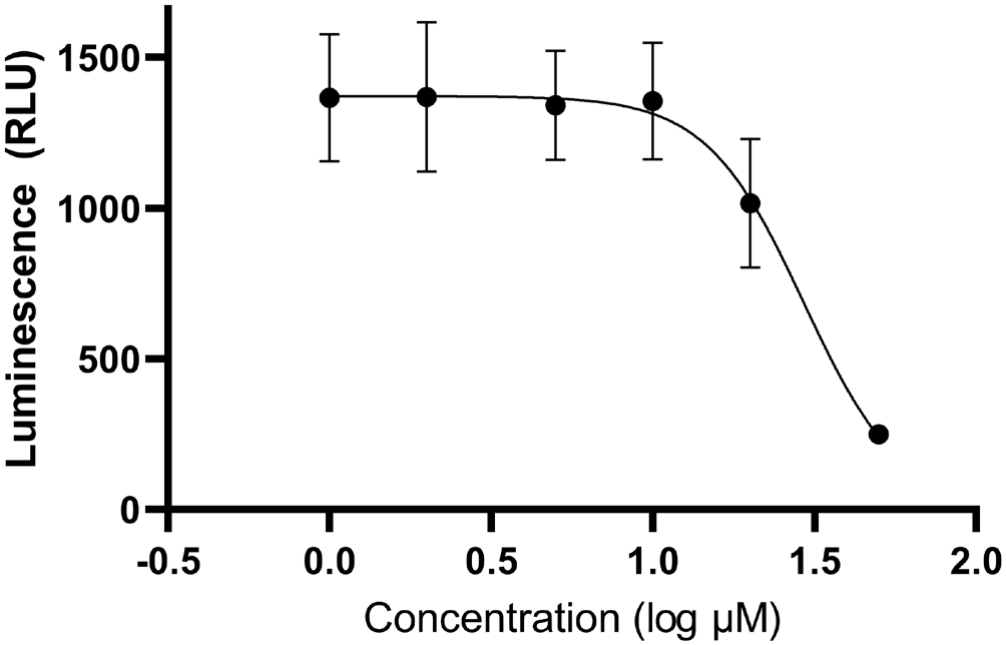
Luminescence (relative luminescence units) against concentration (log μM) of peptide inhibitor 2_3. The IC50 value for this PI was estimated to be 23.59 μM using 4‐parameter logistic regression. Four biological replicates were tested for each concentration. Data represent mean ± SEM.

Table 2 Contains IC50 values and 95% confidence intervals for all tested peptide inhibitors. For 3 (1_1, 1_4 and 2_6) peptide inhibitors, either the upper or lower confidence interval values could not be calculated due to substantial deviation between data points. IC50 values ranged from 20.81 μM for PI 2_8 to 57.19 μM for PI 1_4.

**TABLE 2:** IC50 values in μM for each of the studied novel peptide inhibitors.

Compound	IC50 for HEK293T (μM)	95% CI (μM)
1_1	43.31	34.98 to *
1_2	43.99	34.10 to 51.05
1_3	42.45	31.93 to 55.55
1_4	57.19	* to 179.8
1_5	24.21	20.17 to 29.01
2_1	24.29	19.67 to 30.13
2_2	29.30	23.84 to 36.58
2_3	23.59	17.77 to 30.41
2_4	27.65	19.13 to 39.34
2_5	21.91	17.05 to 28.30
2_6	25.65	* to 32.89
2_7	20.81	15.36 to 27.24
2_8	27.39	22.45 to 34.05
2_9	24.97	1.41 to 29.64

*Note:* * Complete CI ranges could not be established due to excessive data point variance.

### 3.3 Assessment of Peptide Inhibitor Cytotoxicity—Flow Cytometry

**FIGURE 7:**
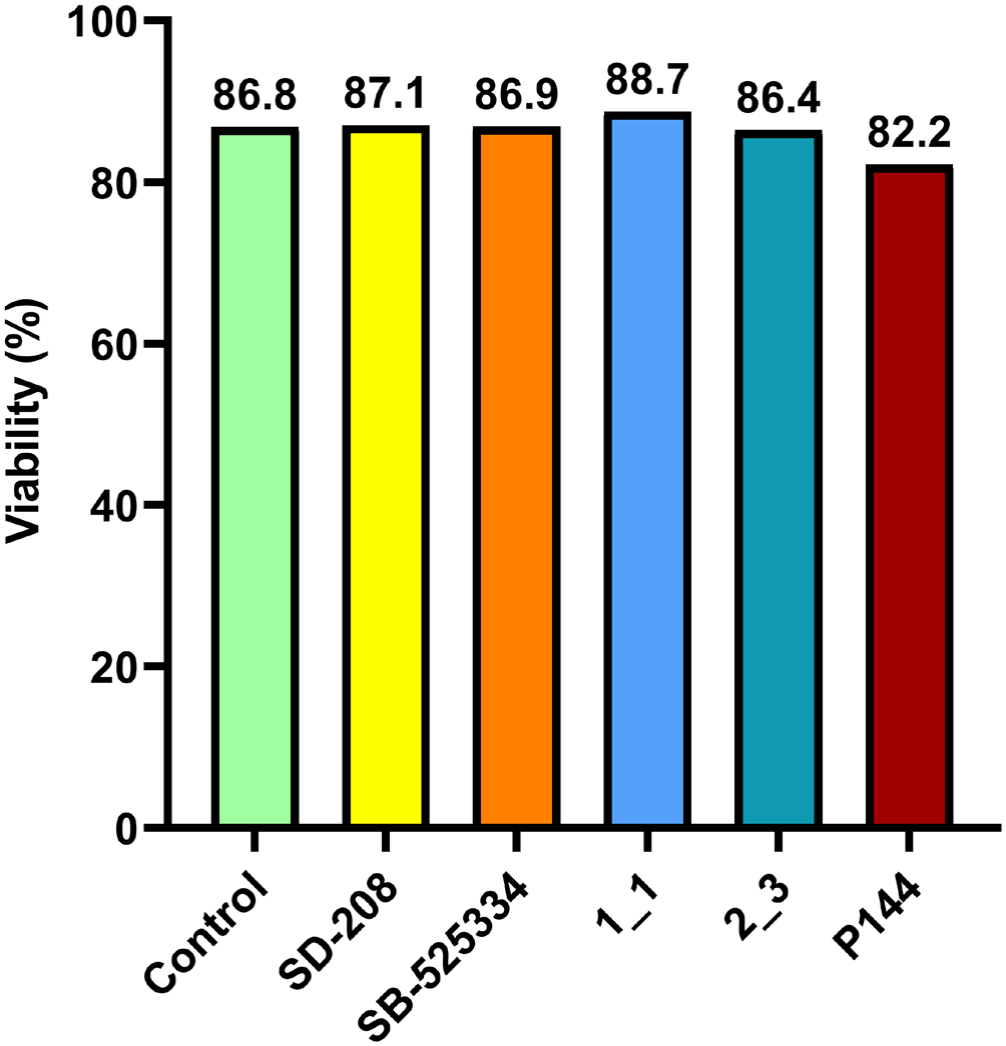
Viability of HEK293T cells incubated with 20 μM TGFBRI/TGFBRII inhibitors for 24 h. Compared to the control sample, neither SD‐208 nor SB‐525334, or the novel peptide inhibitors affected cell viability negatively. Control—untreated HEK293T cells; SD‐208—cells incubated with 20 μM SD‐208; SB‐525334—cells incubated with 20 μM SB‐525334; 1_1—cells incubated with 20 μM novel peptide inhibitor 1_1; 2_3—cells incubated with 20 μM novel peptide inhibitor 2_3; P144—cells incubated with 20 μM P144.

A series of measurements of the impact of SD‐208, SB‐525334, PI 1_1, PI 2_3 and P144 (a commercially available TGFBRI peptide inhibitor) [30] on the viability of HEK293T cells was also performed using flow cytometry. Compared to negative controls (untreated cells), at a concentration of 20 μM, none of the tested inhibitors reduced cell viability to a statistically significant degree. Figure 7 shows the impact of each of the tested compounds on the viability of HEK293T cells.

## 4 Discussion

TGF‐β receptors were found to be mutated in many types of cancer, in which they lead to dysregulation of TGF‐β signalling. Biallelic inactivation of TGFBRII and mutations in the gene encoding TGFBRI occur in kidney and lung cancers [31]. TGFBRII mutations are frequently found in tumours with microsatellite instability [32]. Decreased expression of TGFBRI or TGFBRII was found frequently in lung, kidney, gastric, prostate and bladder cancers [33, 34, 35].

In the context of kidney cancer, it was found that TGF‐β regulates the expression of several microRNAs involved in the progression of renal cell carcinoma (RCC) [36]. TGF‐β was shown to regulate the proliferation of RCC cells—such as those of the Caki‐2 and 786‐O cell lines—through modifying the expression of oncogenic regulators, including ETS1 [37].

In lung cancer, significantly increased TGF‐β expression levels in patients have been shown to be an indicator of decreased survival rates [38]. Furthermore, TGF‐β increases the expression levels of EMT‐inducing transcription factors, such as Snail1, Snail2 and ZEB1/2 [39]. The deep involvement of TGF‐β in kidney and lung cancer development and progression emphasises the potential of TGFBRI/TGFBRII inhibitors as immunotherapeutic agents. TGFBR inhibition may be a valuable strategy for the treatment of several fibrosis‐related and neoplastic disorders [40, 41].

This has prompted researchers to develop multiple types of TGF‐β antagonists for potential therapeutic use. Several TGF‐β antagonists have been in research for anti‐cancer therapies since the early 2000s [42], but so far, none have been accepted for clinical use by the FDA (U.S. Food and Drug Administration) or the EMA (European Medicines Agency). Many such inhibitors have even reached the stage of clinical trials, such as Galunisertib (LY2157299). Despite the drug having an acceptable safety profile and having been shown to improve chemotherapy responses in triple‐negative breast cancer [43], it failed to meaningfully improve patient outcomes in a phase II trial for the treatment of hepatocellular carcinoma [44]. Consequently, its development was discontinued in 2020 [45].

Despite no TGF‐β antagonists suitable for clinical use having been successfully developed, numerous strategies for TGF‐β inhibition are being researched, differing in the elements of the TGF‐β signalling pathway they target. Small molecule TGFBRI kinase inhibitors, such as vactosertib, which is currently undergoing early‐stage clinical trials, prevent the phosphorylation of R‐SMADS [46]. Several monoclonal antibodies, such as fresolimumab, which neutralises mature TGF‐β1/2/3 [47] or LY3022859, which binds TGFBRII [48], are also undergoing early clinical trials. Soluble TGF‐β receptor traps composed of the extracellular domains of TGF‐β receptors, such as bintrafusp alpha (M7824), which traps the TGF‐β1/2/3 ligands, are also under development [49]. However, despite numerous TGF‐β antagonists being researched, very little attention is given to peptide inhibitors of the TGFβRI/TGFβRII/TGFβ complex.

We hypothesised that peptides derived from the TGFβRI/TGFβRII/TGFβ binding complex would have biologically significant inhibitory effects on the TGF‐β signalling pathway, with no significant cytotoxicity. In our study, we show preliminary results of a series of in silico designed peptide inhibitors on the HEK293T CAGA12‐Luc/TK Renilla cell line, which was designed to assess TGF‐β receptor activity. Upon selection of the most biologically active PIs, the docking between peptides and receptors will be more closely examined using molecular docking software and other bioinformatic methods. On this basis, the chances of improving the activity of said peptides through elongation, shortening, or mutagenesis will be estimated. The biological activity of these newly designed peptides will then again be assessed using molecular methods. This process can be rapidly repeated until sufficiently active peptides are discovered.

In the luminescence‐based assays we conducted, all of the PIs tested exhibited statistically significant inhibitory activity against TGF‐β receptors at concentrations of 20 μM and 50 μM.

At 50 μM, the highest concentration we tested, TGF‐β receptor activity was reduced by 80% on average, compared to positive controls. Critically, at 50 μM, there were no statistically significant differences between the inhibitory effects of peptides 2_5, 2_6 and 2_7 and SD‐208 at 20 μM. These results prove that the novel PIs are biologically active inhibitors of the TGFβRI/TGFβRII/TGFβ1 complex. Additionally, peptides can achieve higher target specificity and lower cytotoxicity than small molecule‐based agents [50], which is especially important for the development of more efficacious and safer immuno‐oncological therapeutics. To underline this point, none of the PIs tested in cytometric assays of apoptosis at 20 μM reduced cell viability compared to untreated controls.

The IC50 values for our PIs ranged from 20.81 μM for PI 2_8 to 57.19 μM for PI 1_4. Importantly, for three of the PIs (1_1, 1_4 and 2_6), complete confidence interval (CI) ranges could not be established due to significant data scatter, which limits the interpretability of the results for these PIs. However, due to the high amount of tested compounds for which complete Cis were established successfully, this does not constitute a significant issue for the interpretability of the results in totality. The IC50s for the small molecule inhibitors we utilised are far lower—at 94 nM for SB‐431542 [51] and 48 nM for SD‐208 [52], respectively. However, this difference is partially negated by the disparity in molecular weight between peptide inhibitors and small particle agents such as SB‐431542 and SD‐208. While the molar concentrations used to achieve biological effects are far higher for PIs, the actual therapeutic mass ultimately needed to elicit inhibition of signalling pathways would ultimately not differ as greatly. There is no published data on IC50s for TGFBR PIs, but for peptide‐based agents in general, an IC50 in the range of 20 to 40 μM indicates the presence of biological activity, albeit not very robust [53].

Low cytotoxicity in conjunction with significant biological activity exhibited by our PIs is an attractive characteristic for potential immunomodulating pharmacological agents.

In general, very little data on the efficacy of TGFBRI/TGFBRII peptide inhibitors has been published; thus, direct comparisons are difficult. Additionally, the results of our study constitute preliminary research and will be complemented by several more assays in the near future.

P17 is one of the most widely studied TGF‐β peptide inhibitors. It has been shown to significantly decrease the expression of CTGF (connective tissue growth factor), which is a mediator of TGF‐β signalling [54]. P17 has also exhibited the ability to inhibit Treg activity both in vitro and in vivo and inhibit the suppressive effects of Tregs on effector T cells in vitro. Additionally, the peptide improved antitumor immunogenicity in mice. Lastly, P17 was shown to improve CD4+ T‐cell proliferation in mice implanted with EG.7‐OVA tumours [55]. The novel peptide inhibitors described in this pilot study have not yet been tested in vivo—and as such, their effects cannot be compared to data published on P17; however, the effects of our inhibitors on T‐cell exhaustion and the TME will be studied in the near future.

Aside from the peptide inhibitors described in this study, only one peptide‐based direct inhibitor of the TGFB/TGFBRI/TGFBRII complex is currently being researched. Yuan et al. reported in 2022 that a peptide derived from Klotho (a protein which plays a critical role in the pathogenesis of chronic kidney disease) limits renal fibrosis and preserves kidney function in mouse models of kidney disease. The inhibitor, named KP1, was shown to bind TGFBRII, thus blocking signal transduction and activation of the SMAD and MAPK pathways [56]. This study is not directly comparable to our current research, which focuses primarily on chronic inflammatory diseases and immuno‐oncology, rather than organ fibrosis. Nevertheless, the study published by Yuan et al. reinforces the hypothesis that TGFBR inhibition may limit cancer immune exclusion by decreasing the degree of tissue fibrosis.

Thus far, peptide inhibitors for only two out of the five identified TGFBRI/TGFBRII interface regions were synthesised and tested via the assays specified above. Functional research on cancer cell lines and CD4+, CD8+ lymphocytes will be carried out as part of the current project to ultimately confirm the immunomodulatory characteristics of the novel peptide inhibitors.

In particular, we aim to assess the influence of the PIs on MAPK and SMAD pathway protein expression and phosphorylation via immunoblotting, determine the protein‐receptor binding constant for the most biologically active PI via microscale thermophoresis (MST), assess the effects of our PIs on A498, 786‐O, Caki‐1 and Caki‐2 cancer cell lines via MTT assays, clonogenic assays, cytometric measurements and RNA‐seq—and finally evaluate the influence of PIs on CD4+/CD8+ T‐cell exhaustion with the use of cytokine panels and immunoblotting.

As of late, much research in the field of cancer immunotherapy has been focused on finding combinations of immunotherapeutics with either traditional antitumor therapies or additional immunotherapeutic agents, rather than using immunotherapy as a monotherapy [57]. Due to the robust influence of TGF‐β on the TME, effective and safe TGF‐β inhibitors can serve as a supplementary therapy used in conjunction with conventional immunotherapies, which are commonly hampered by lymphocyte depletion [58]. Aside from limiting fibrosis and modulating the TME, TGF‐β inhibitors can also block lymphocyte depletion, drastically improving the efficacy of widely used adoptive cell antitumour therapies such as tumour‐infiltrating lymphocyte (TIL) therapy, engineered T cell receptor (TCR) therapy or chimeric antigen receptor (CAR) T cell therapy [59].

Clinical translation of peptide‐based therapeutics is an ongoing challenge in the field of biologics. Despite promising preliminary data, most therapeutic peptides do not successfully pass clinical trials. An example of this is cliengitide (a peptide‐based integrin antagonist), which was tested against glioblastoma and discontinued after it failed in phase III randomised clinical trials [60]. However, there do exist examples of success in this field, such as carfilzomib, which is a second‐generation proteasome peptide inhibitor. In 2012, the peptide received FDA approval for use in patients with refractory multiple myeloma [60]. As of 2023, over 80 peptides have been approved by either the FDA or EMA for treatment of infectious, cardiovascular, dysmetabolic and neoplastic diseases [60]. Concurrently, hundreds more peptides are being developed in preclinical studies and undergoing clinical studies. Over the past few years, a significant rise in the number of patents and publications in this field has been described, which points towards an expansion in interest, investment and research efforts.

Given the promising preliminary results, the PIs described here will be developed and tested further using the methods described above. If one or more of the PIs reach the stage of clinical trials, they have the potential to become a promising therapeutic strategy in the treatment of various pathologies characterised by dysregulated TGF‐β signalling, including fibrotic diseases, asthma, COPD and certain cancers, especially immune‐cold and immune‐excluded cancers. In fibrotic diseases, inhibition of the TGF‐β receptor can help prevent or reverse tissue fibrosis. In asthma and COPD, TGF‐β receptor inhibition may mitigate airway remodelling and inflammation, leading to improved lung function and symptom relief. Additionally, TGF‐β receptor inhibition can mitigate tumour progression and immune evasion in cancers by inhibiting EMT‐inducing cellular signalling and preventing fibrotic barriers from forming and causing immune exclusion of tumours. Thus, TGF‐β receptor peptide inhibitors offer significant therapeutic potential across multiple diseases, with the ability to limit disease progression and improve patient outcomes.

## 5 Limitations

The study described here constituted preliminary research; as such, the results should be verified by further assessments of biological activity and toxicity. The influence of the PIs on expression and phosphorylation of the protein components of both Smad‐dependent and Smad‐independent TGF‐β signalling via immunoblotting (Western blotting) needs to be carried out. This would constitute evidence that the PIs affect TGF‐β signalling at an intracellular level. To evaluate the effects of the novel inhibitors on cancer cells, several tests need to be carried out on cancer cell lines, in particular lung and kidney cancer lines. These assays include, but are not limited to: MTT assays, clonogenic assays, migration and invasion tests (scratch tests and cytometric measurements of cancer cell apoptosis. Finally, to assess the effects of the PIs on CD4+/CD8+ T‐cell exhaustion, the protein expression profiles of T cells incubated with TGF‐β and the novel PIs should be studied with the use of cytokine panels and immunoblotting.

As the current study is preliminary and based solely on in vitro methods, the range of experimental interpretations is limited. In later stages of research on our PIs, more molecular and in vivo methods need to be employed to assess the receptor binding affinities of our inhibitors, their pharmacodynamics, toxicity and safety profiles.

Additionally, peptide‐based therapeutics face a range of challenges related to scalability, stability and delivery mechanisms. The manufacturing processes required for biologics are far more complex and costly than those used in small molecule manufacturing, as living cells or organisms need to be utilised. Over the last few decades, the cost of biologics manufacturing did decrease significantly but remains high compared to other classes of pharmaceuticals. The regulations related to biologics manufacturing are also complex and strict, making production scalability even more challenging. Biologics are also susceptible to proteases, aggregation and denaturation, which can lead to a loss of biological activity or even harmful immune reactions. What is more, biologics are also very sensitive to temperature fluctuations, requiring precise control and monitoring of storage conditions. Administration is another significant limitation of peptide‐based therapeutics. Most biologics are currently administered intravenously, which increases costs and lowers patient compliance. Oral, topical and inhaled biologic formulations are also being developed, but those routes of administration face their own challenges, such as overcoming physiological barriers, requiring higher solution concentrations, or requiring specialised devices.

## 6 Conclusions

In conclusion, our peptide inhibitors of TGFBRI/TGFBRII have shown activity in vitro, as demonstrated in the luminescence‐based assays, while exhibiting no cytotoxicity in cytometric assessments. Thus, our results suggest that the novel PIs described here may be of potential interest in the treatment of fibrotic disorders, chronic inflammatory diseases, or certain neoplastic cancers.

The novel PIs will be further refined in silico and their biological efficacy tested via assays carried out on cancer cell lines and CD4+/CD8+ T cells.

## Author Contributions

**Jacek Plichta:** conceptualization (supporting), data curation (equal), formal analysis (equal), investigation (lead), methodology (supporting), software (supporting), visualization (lead), writing – original draft (lead), writing – review and editing (equal). **Michał Karbownik:** formal analysis (equal), methodology (equal), resources (equal), software (equal), validation (equal), visualization (supporting), writing – review and editing (equal). **Piotr Kuna:** conceptualization (equal), funding acquisition (equal), project administration (equal), supervision (equal), validation (equal), writing – review and editing (equal). **Michał Panek:** conceptualization (equal), funding acquisition (equal), methodology (equal), project administration (equal), supervision (equal), validation (equal), writing – review and editing (equal).

## Ethics Statement

This study received approval from the bioethical committee of the Medical University of Lodz (approval no RNN/186/24/KE).

## Conflicts of Interest

The authors declare no conflicts of interest.

## Supporting information


Figures S1‐S12.

Table S1‐S4.

## Data Availability

The original contributions presented in this study are included in the article/Supporting Information. Further inquiries can be directed to the corresponding author. Raw data (spectrophotometric and cytometric measurement output) files are available on request from the corresponding author.
