# Supplementary material for: In Silico–Designed TGFβRI/TGFβRII Receptor Complex Peptide Inhibitors Exhibit Biological Activity In Vitro
Source: J Cell Mol Med. 2025 Apr 17;29(8):e70548. doi: 10.1111/jcmm.70548 (PMC12005349; doi:10.1111/jcmm.70548)
Supplement: Supplementary file 1 — Figures S1‐S12. [file JCMM-29-e70548-s002.pdf]

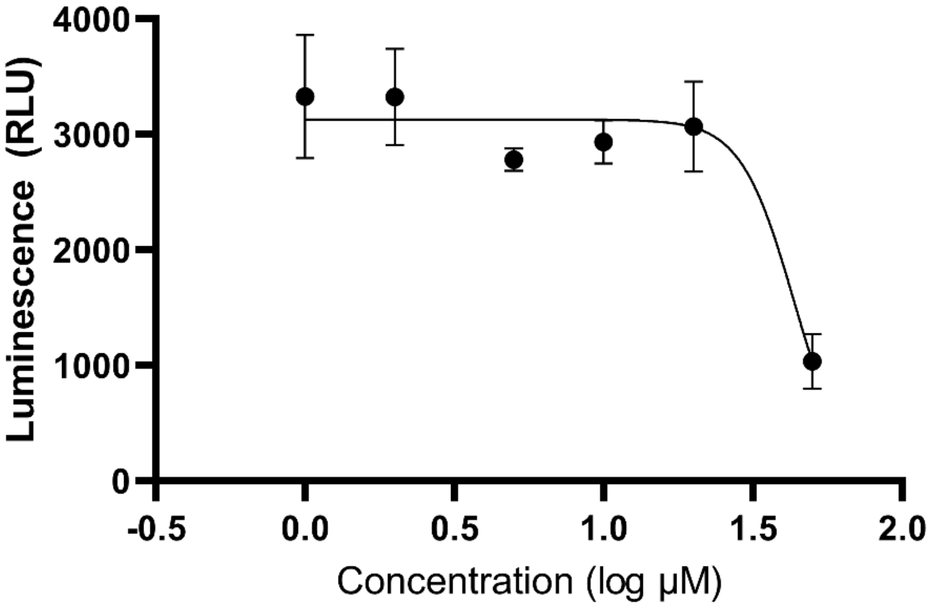

Figure S1. Luminescence (relative luminescence units) against concentration (log μM) of peptide inhibitor 1_1. The IC50 value for this PI was estimated to be 43.31 μM using 4-parameter logistic regression. 4 biological replicates were tested for each concentration. Data represent mean ± SEM.

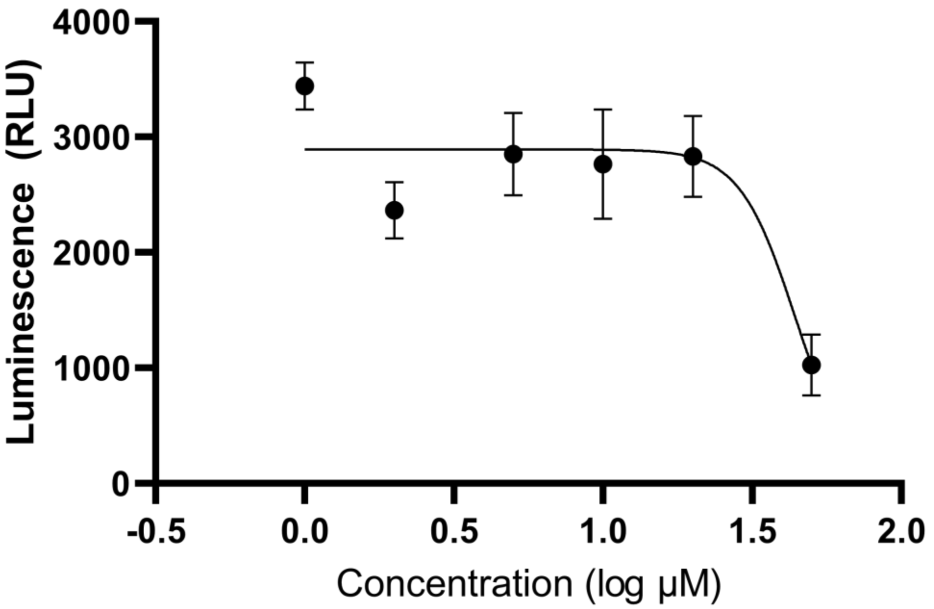

Figure S2. Luminescence (relative luminescence units) against concentration (log μM) of peptide inhibitor 1_2. The IC50 value for this PI was estimated to be 43.39 μM using 4-parameter logistic regression. 4 biological replicates were tested for each concentration. Data represent mean ± SEM.

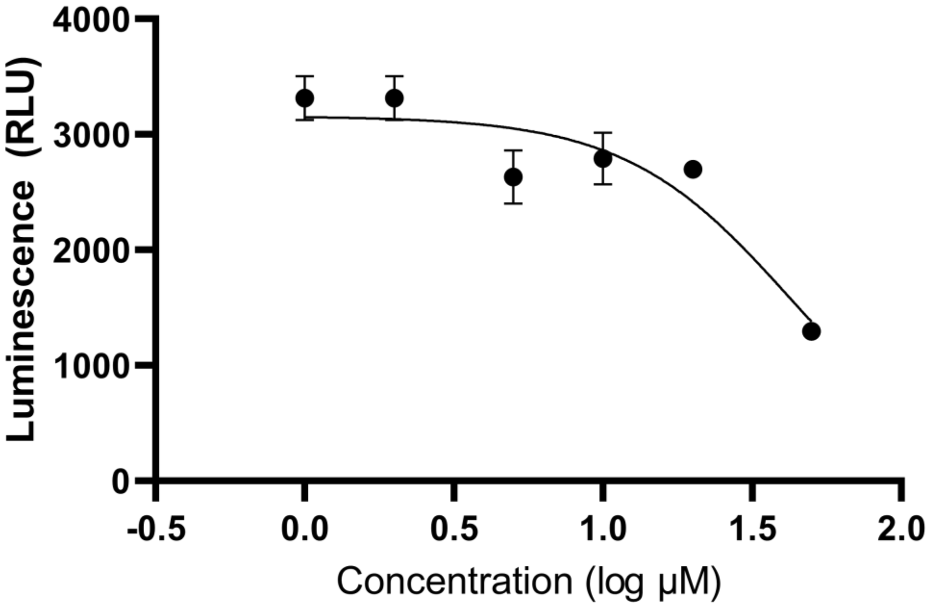

Figure S3. Luminescence (relative luminescence units) against concentration (log µM) of peptide inhibitor 1_3. The IC50 value for this PI was estimated to be 42.45 µM using 4-parameter logistic regression. 4 biological replicates were tested for each concentration. Data represent mean ± SEM.

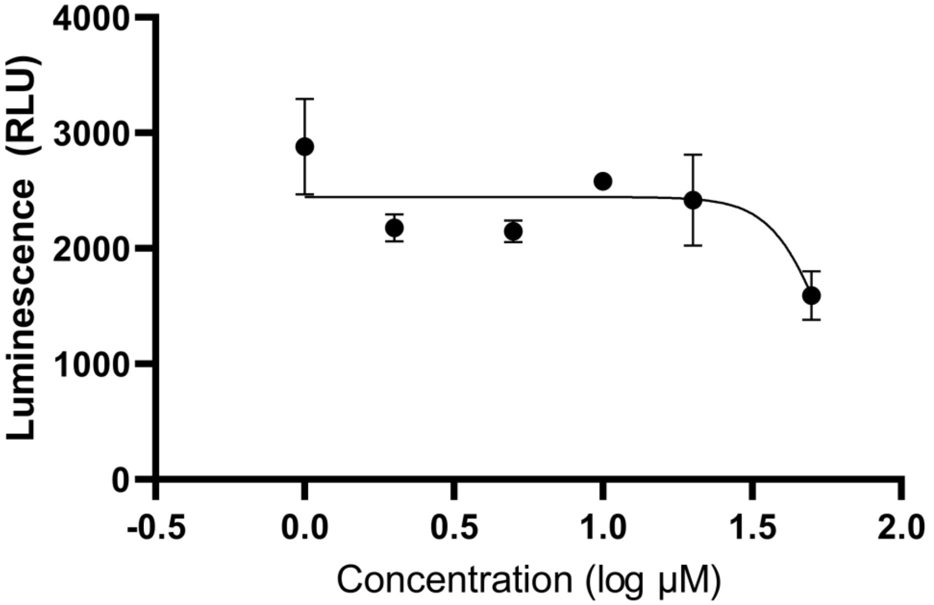

Figure S4. Luminescence (relative luminescence units) against concentration (log µM) of peptide inhibitor 1_4. The IC50 value for this PI was estimated to be 57.19 µM using 4-parameter logistic regression. 4 biological replicates were tested for each concentration. Data represent mean ± SEM.

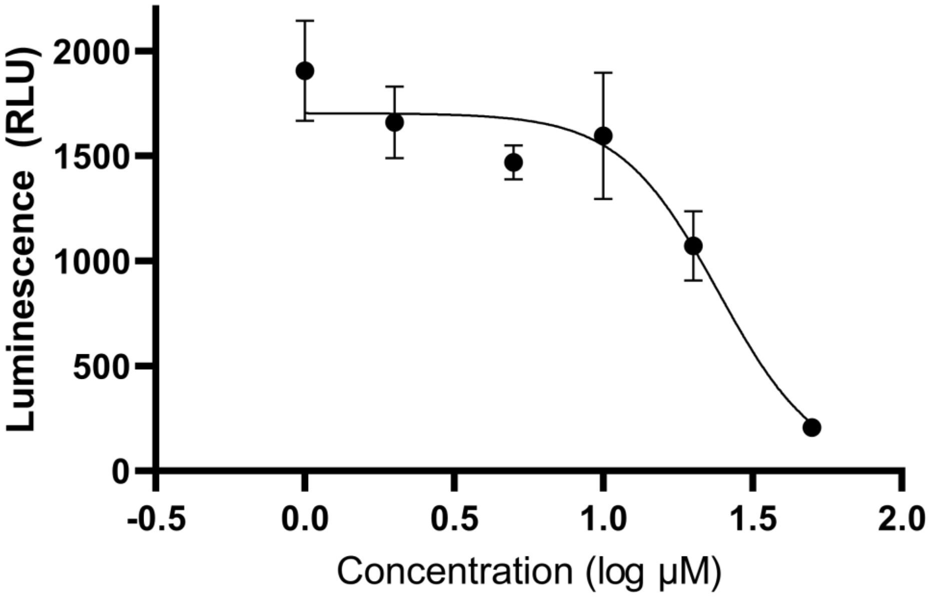

Figure S5. Luminescence (relative luminescence units) against concentration (log μM) of peptide inhibitor 2_1. The IC50 value for this PI was estimated to be 24.29 μM using 4-parameter logistic regression. 4 biological replicates were tested for each concentration. Data represent mean ± SEM.

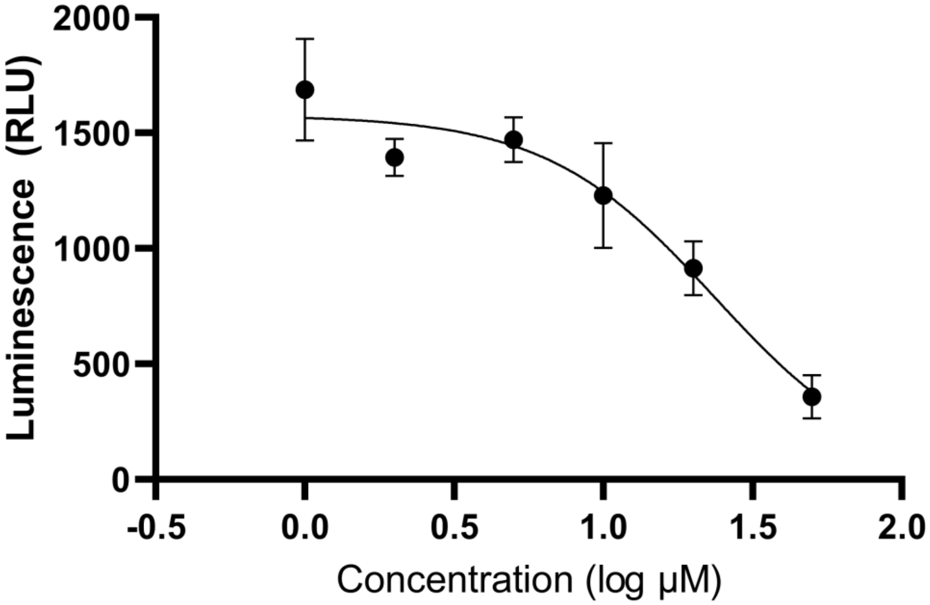

Figure S6. Luminescence (relative luminescence units) against concentration (log μM) of peptide inhibitor 2_2. The IC50 value for this PI was estimated to be 29.30 μM using 4-parameter logistic regression. 4 biological replicates were tested for each concentration. Data represent mean ± SEM.

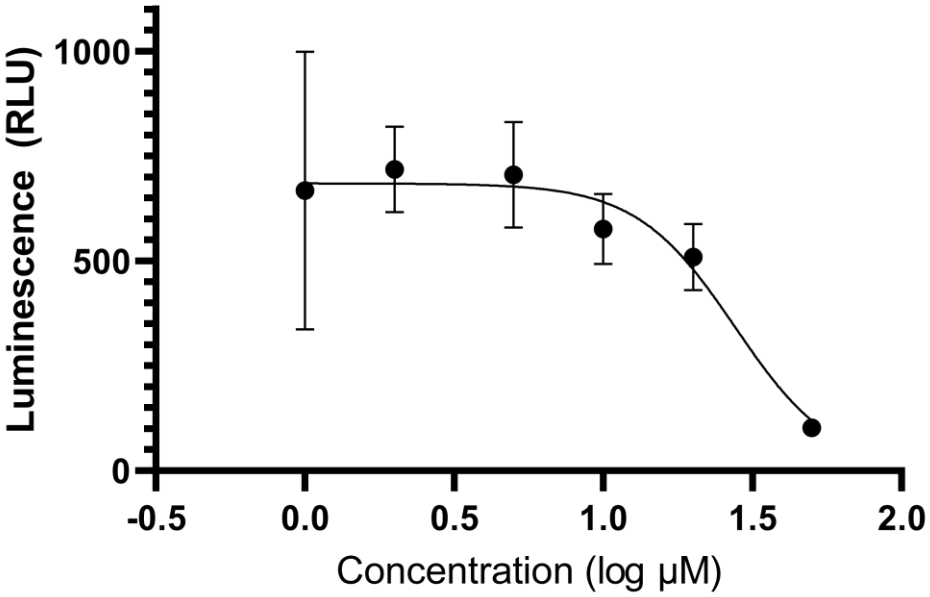

Figure S7. Luminescence (relative luminescence units) against concentration (log μM) of peptide inhibitor 2_4. The IC50 value for this PI was estimated to be 27.65 μM using 4-parameter logistic regression. 4 biological replicates were tested for each concentration. Data represent mean ± SEM.

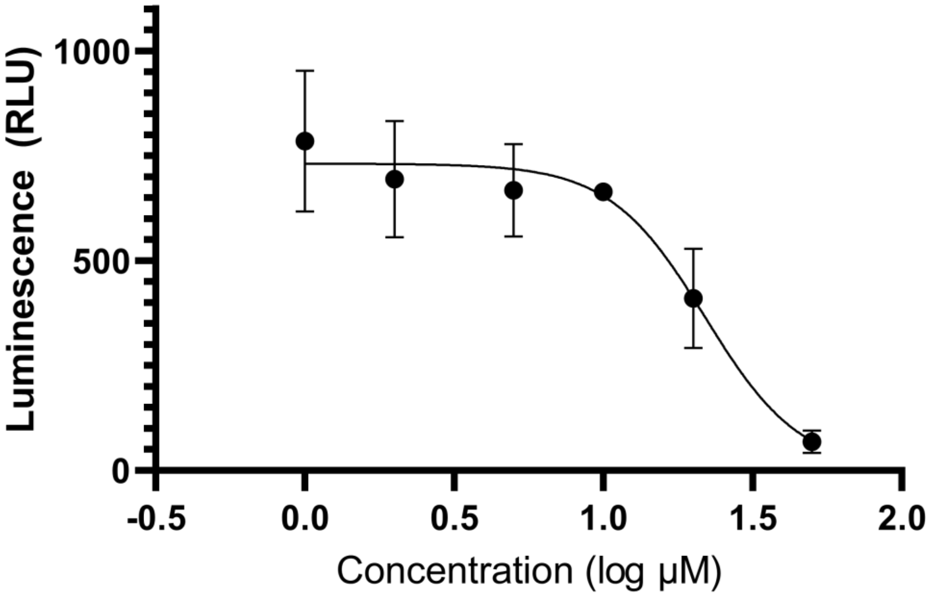

Figure S8. Luminescence (relative luminescence units) against concentration (log μM) of peptide inhibitor 2_5. The IC50 value for this PI was estimated to be 21.91 μM using 4-parameter logistic regression. 4 biological replicates were tested for each concentration. Data represent mean ± SEM.

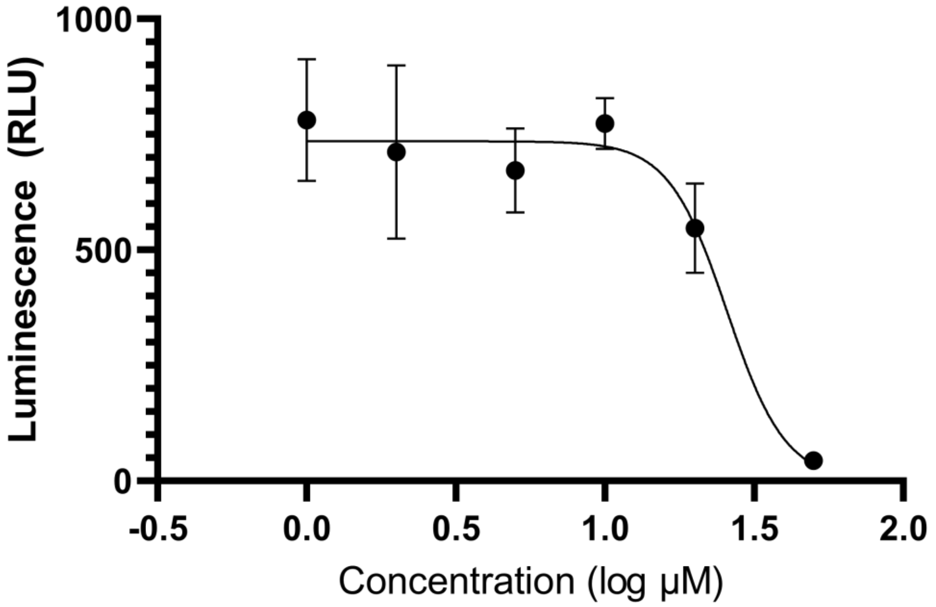

Figure S9. Luminescence (relative luminescence units) against concentration (log µM) of peptide inhibitor 2_6. The IC50 value for this PI was estimated to be 25.65 µM using 4-parameter logistic regression. 4 biological replicates were tested for each concentration. Data represent mean ± SEM.

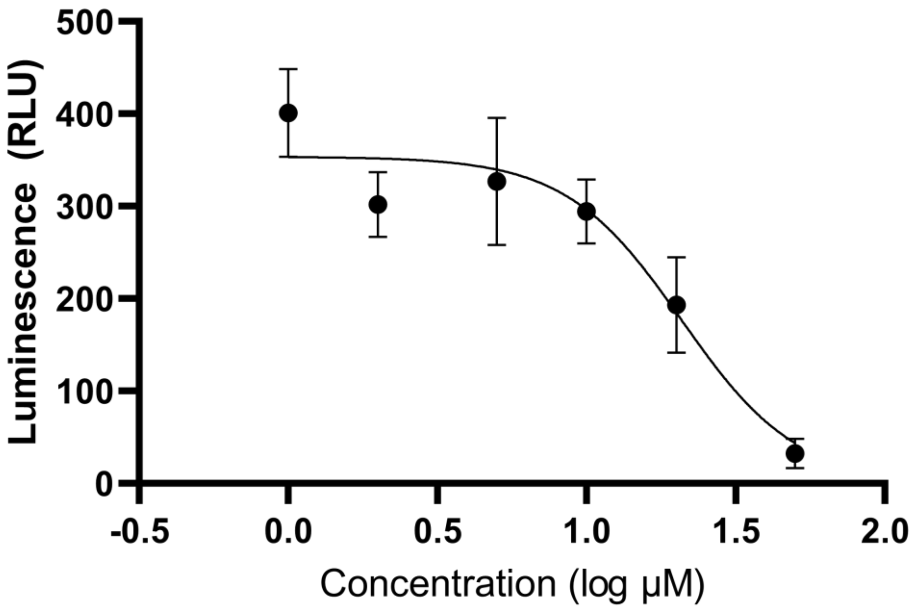

Figure S10. Luminescence (relative luminescence units) against concentration (log µM) of peptide inhibitor 2_7. The IC50 value for this PI was estimated to be 20.81 µM using 4-parameter logistic regression. 4 biological replicates were tested for each concentration. Data represent mean ± SEM.

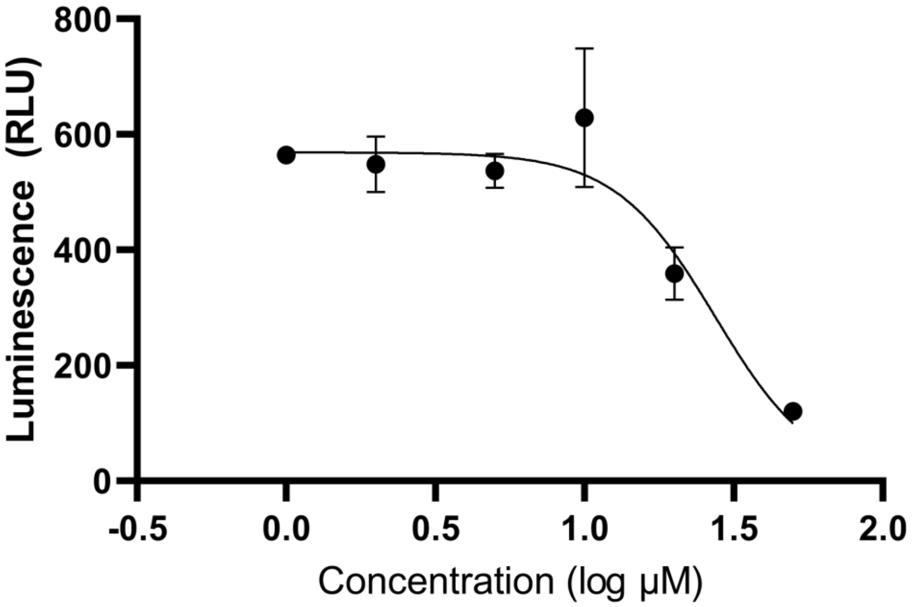

Figure S11. Luminescence (relative luminescence units) against concentration (log μM) of peptide inhibitor 2_8. The IC50 value for this PI was estimated to be 27.39 μM using 4-parameter logistic regression. 4 biological replicates were tested for each concentration. Data represent mean ± SEM.

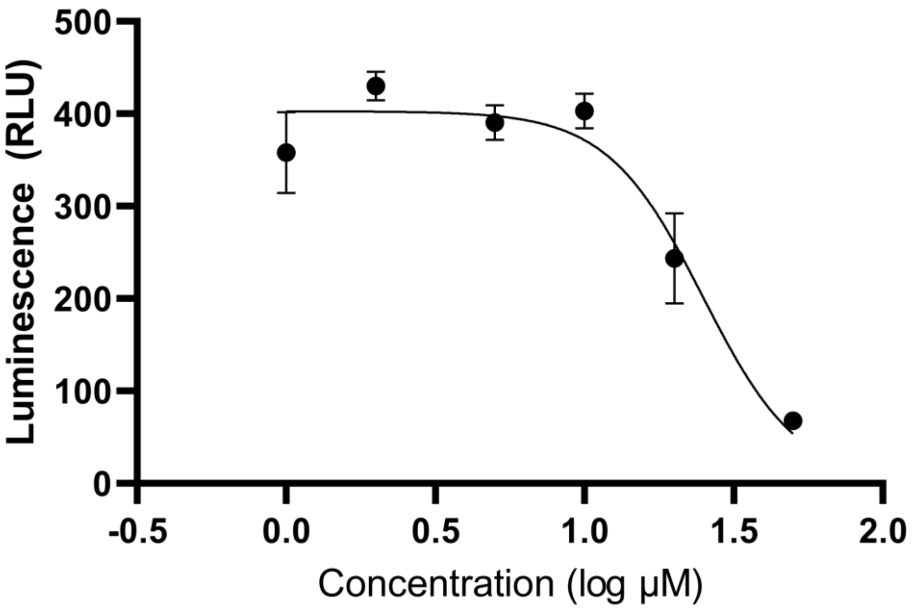

Figure S12. Luminescence (relative luminescence units) against concentration (log μM) of peptide inhibitor 2_9. The IC50 value for this PI was estimated to be 24.97 μM using 4-parameter logistic regression. 4 biological replicates were tested for each concentration. Data represent mean ± SEM.
